# Economic Design of Solar-Driven Membrane Distillation Systems for Desalination

**DOI:** 10.3390/membranes11010015

**Published:** 2020-12-24

**Authors:** Yih-Hang Chen, Hwo-Gan Hung, Chii-Dong Ho, Hsuan Chang

**Affiliations:** Department of Chemical and Materials Engineering, Tamkang University, New Taipei City 25137, Taiwan; abcd91082@hotmail.com (H.-G.H.); nhchang@mail.tku.edu.tw (H.C.)

**Keywords:** solar energy, desalination, optimization, air gap membrane distillation, direct contact membrane distillation, vacuum membrane distillation

## Abstract

Solar-driven membrane distillation (SDMD) for desalination is a feasible method to solve water and energy resource issues. The design and operation of SDMD is different from continuous and steady state processes, such as common chemical plants, due to the intermittent and unpredictive characteristics of solar radiation. Employing the steady state and dynamic simulation models developed on the platform of Aspen Custom Modeler^®^, this paper presents a two-stage design approach for the SDMD systems using different types of membrane distillation configurations, including AGMD (air gap MD), DCMD (direct contract MD) and VMD (vacuum MD). The first design stage uses the steady state simulation model and determines equipment sizes for different constant-value solar radiation intensities with the objective of minimizing total annual cost. The second design stage is implemented on the SDMD systems with process control to automatically adjust the operating flow rates using the dynamic simulation model. Operated with the yearly solar radiation intensity of Taiwan, the unit production costs (UPCs) of the optimal SDMD systems using AGMD, DCMD, and VMD are $2.71, 5.38, and 10.41 per m^3^ of water produced, respectively. When the membrane unit cost is decreased from $90/m^2^ to $36/m^2^, the UPC of the optimal solar-driven AGMD system can be reduced from $2.71/m^3^ to $2.04/m^3^.

## 1. Introduction

Climate change due to greenhouse effects has caused the re-distribution of worldwide water resources [1,2,3]. The mostly adopted desalination technology for producing potable water from salted water or seawater is reverse osmosis. This mature technology is driven by a high-pressure difference across the membrane and is operated with electric energy [4,5,6]. Scientists have paid much attention to an environmental way to generate desalinated water. Therefore, combining solar thermal energy, which is renewable, and membrane distillation technology, which can be operated using low-grade thermal energy, has been extensively studied in recent years [7,8]. This combined system resolves both water resource and energy resource problems and contributes the sustainable development [9,10,11,12,13,14].

The four basic configurations of membrane distillation are direct contact (DCMD), air gap (AGMD), vacuum (VMD), and sweeping gas membrane distillation (SGMD) [11]. In a comparative assessment of full-scale DCMD modules with various single- and multi-channel designs, Winter et al. [15] concluded that, in addition to flux and thermal energy consumption, economic consideration must be included.

For the solar-driven membrane distillation (SDMD) desalination, Banat and Jwaied [16] designed solar-driven AGMD systems for daily water production rates of 100 L and 500 L and the costs are $15/m^3^ and $18/m^3^, respectively. Recently, an economic optimization of solar powered SGMD desalination system has been reported [17]. The study concluded that membranes and thermal collectors are the main contributors to capital cost and the water production cost is $85/m^3^. Miladi et al. [18] focused on the energy performance of a solar-driven VMD. Rather than design or optimize the systems for yearly operation, these studies [17,18] were conducted using a specified daily solar radiation profile.

The dynamic simulation and operation of solar MD desalination system are important due to the time variant nature of solar radiation. Chang et al. [13,19] built mathematical models of all the units involved in the system and discussed the operation and control issues of a solar-driven AGMD desalination plant. Chen et al. [20] and Chang et al. [21] discussed the design and control of the solar-driven AGMD desalination plant. Gil et al. [22] discussed the performance of four control schemes for a pilot solar MD facility. Bendevis et al. [23] proposed a bang-bang controller for a solar thermal MD system.

As the SDMD system is powered by the solar radiation, both design and operation of the system must take into account the unpredictive and intermittent feature of solar radiation. This work presents a systematic two-stage design approach for the SDMD system. The design considers actual radiation intensity, utilizes a dynamic simulation model, and includes a control system for continuous operation. In the following sections, the process description and simulation model are presented first, followed by the explanation of the process control, the introduction of the two-stage design procedure, and the results of the design for the solar radiation of Taiwan.

## 2. Process Description and Modeling

This section includes four parts. First, the overall SDMD systems are illustrated. Secondly, the mathematic models of the individual units are explained. The third part presents the validation of the mathematic models of membrane distillation modules using experimental operation conditions and results from literature. The last part gives the simulation results of representative cases for the overall SDMD systems.

### 2.1. Process Description

The major process units of a SDMD system for desalination are the solar collector and the membrane distillation module. The processes employing AGMD, DCMD, and VMD are different and explained in detail in this section.

#### 2.1.1. Air Gap Membrane Distillation System for Desalination (AGMD)

**Figure 1:**
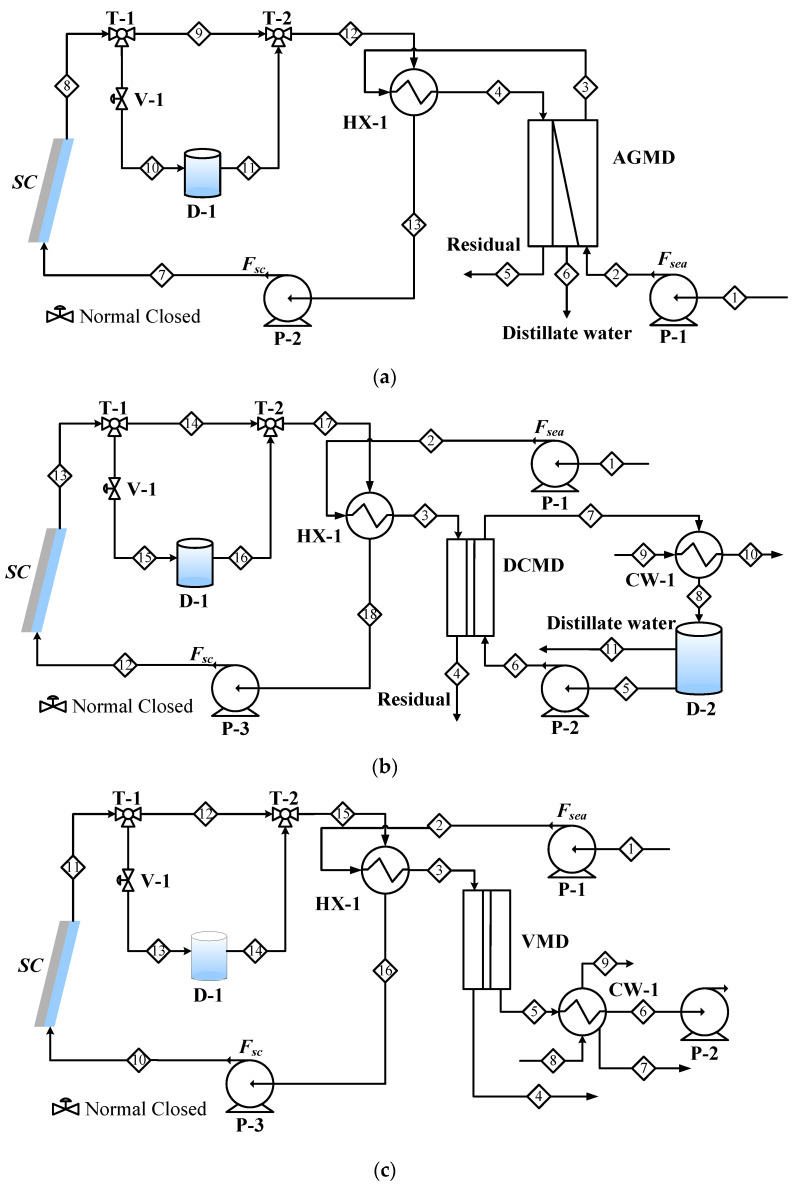
Process flow diagrams of solar-driven (**a**) AGMD, (**b**) DCMD, (**c**) VMD systems for desalination.

The process flow diagram of the solar-driven AGMD desalination system, which comprises a membrane distillation subsystem and a solar subsystem, is shown in Figure 1a. In the membrane distillation subsystem, the seawater (Stream 1) is pumped into the cold side of AGMD module. The AGMD module consists of a hot water flow channel, a cold water flow channel, a hydrophobic porous membrane, and an air gas layer, which is located between the membrane and cold seawater flow channel. In the heat exchanger (HX-1), the cold seawater leaving the MD module (Stream 3) is further heated by the hot circulation water (Stream 12) from the solar subsystem before serving as the hot feed (Stream 4) to the MD module. The amount of water vapor permeated across the porous hydrophobic membrane is determined by the temperature difference between the two sides of the membrane. The distillate water (D) (Stream 6) from the AGMD module is collected and delivered to a storage tank.

In the solar subsystem, water (Stream 7) enters the solar collector (SC) and absorbs the solar radiation. The hot water leaving SC (Stream 8) is partly sent directed to HX-1 (Stream 9) and partly sent to a thermal storage tank (D-1) (Stream 10) before being withdrawn to HX-1 (Stream 11). The split (T-1) is adjusted by a valve (V-1). The overflow design of D-1 tank allows the operation with the same inlet and outlet flowrates. The initial temperature of D-1 is 50 °C. The temperature of D-1 increases with the increase of the radiation energy provided to the solar collector.

#### 2.1.2. Direct Contact Membrane Distillation System for Desalination (DCMD)

The process flow diagram of the solar-driven DCMD desalination system is shown in Figure 1b. Compared to the AGMD system, only the membrane distillation subsystem is different. The operation of DCMD utilizes a pure water stream to serve as the cold stream (Stream 6) to carry out the distillate water from the membrane module. The permeate side outflow (Stream 7) from the MD module is then cooled in a heat exchanger (CW-1) and collected in tank D-2. The overflow from D-2 is the distillate water product. Fresh seawater (Stream 1) is heated by the hot circulation water from the solar subsystem before serving as the hot feed (Stream 3) to the MD module. To avoid the use of expensive construction material for CW-1, it is operated using cooling water instead of seawater.

#### 2.1.3. Vacuum Membrane Distillation System for Desalination (VMD)

The process flow diagram of the solar-driven VMD desalination system is shown in Figure 1c. The solar subsystem is the same as the AGMD and DCMD systems. As VMD is operated under vacuum condition for the permeate side, a vacuum pump (P-2) is needed and the permeated water vapor leaving the MD module (Stream 5) must be cooled and condensed via a heat exchanger (CW-1) to be the distillate water. The hot feed stream arrangement is the same as that of the DCMD system. As explained in Section 2.1.2, CW-1 is operated using cooling water.

### 2.2. Modeling

For the solar-driven AGMD, DCMD, and VMD systems for desalination, the dynamic mass and heat balance equations for each unit equipment are listed in Table 1. For all the units, the model is one-dimensional. The explanations are provided in the following. The details of the models are referred to the previous publications of the authors [13,19,20,21].

#### 2.2.1. Solar Collector

The solar collector consists of an absorption plate and a working fluid channel. The solar collector utilizes an absorption plate to absorb the solar radiation and uses water as the working fluid. We assume well insulation of the solar collector. Therefore, the working fluid of the solar collector is adiabatic. However, the absorption plate of the solar collector contacts with surrounding, the energy will loss due to convection. The assumptions of the model include [19]: (1) The working fluid velocity is uniform in the flow direction. (2) the operating temperature of the working fluid is below 95 °C to avoid phase change in the solar collector. The model consists of the dynamic energy balance equations for the collector and the fluid.

#### 2.2.2. Heat Exchanger

Heat exchangers are of the countercurrent shell and tube type. The model assumptions are: (1) The heat capacity of the working fluid is constant considering the temperature range of operation. (2) The energy loss to the surrounding is negligible. (3) No phase change occurs during operation. The heat capacity of the working fluid and the overall heat transfer coefficient of the heat exchanger are 4180 J/kg-K and 280 W/m^2^-K, respectively. The model consists of the dynamic energy balance equations for the hot fluid and cold fluid.

#### 2.2.3. Thermal Storage Tank

The excess thermal energy during the day time operation is stored in the thermal storage tank (D-1 in Figure 1a). The working fluid is water. The initial residence time and temperature of the tank are 10 min and 25 °C, respectively. By assuming the thermal storage tank is well insulated, the energy loss to the environment is set to be zero. The model assumes there is no phase change of the working fluid. The model consists of the dynamic energy balance equations for the solar collector and the working fluid.

#### 2.2.4. Membrane Distillation Modules

**Figure 2:**
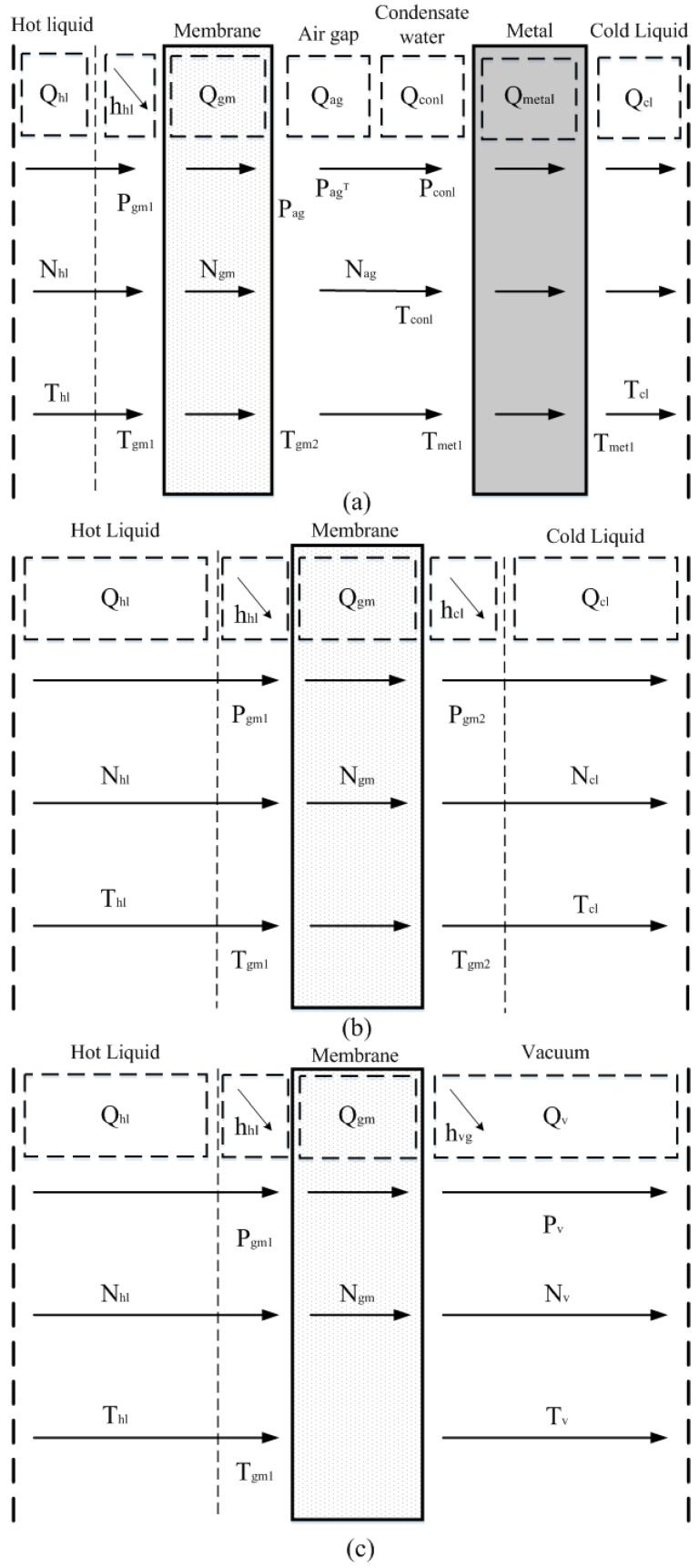
The variable notations of (**a**) AGMD, (**b**) DCMD, (**c**) VMD modules.

The AGMD module consists of hot and cold seawater flow channels, a membrane layer, an air gap layer, and a metal layer. The DCMD module consists of a hot seawater channel, a cold water flow channels, and a membrane layer. The VMD module consists of a hot seawater flow channel, a membrane layer, and a vacuum layer. The detailed drawing of each module and variable notations are shown in Figure 2a–c. The model assumptions of these modules are: (1) The vaporization of water occurs at the interface between the hot fluid and the membrane. (2) The heat and mass transfer across the interfaces can be described using film theory with correlations of heat and mass transfer coefficients. (3) Only water vapor can transfer through the membrane pores. (4) The membrane module operation is adiabatic.

Because of the small holdup volume of the membrane module, steady state models were employed. The model equations for each type of the membrane modules are summarized in Table 1, including the mass balance and energy balance for the fluid of each channel as well as the transmembrane mass flux of water and heat flux. The heat and mass transfer coefficients were estimated using the methods from literature [24,25,26,27,28,29].

### 2.3. Membrane Distillation Model Validation of Membrane Distillation Modules

**Figure 3:**
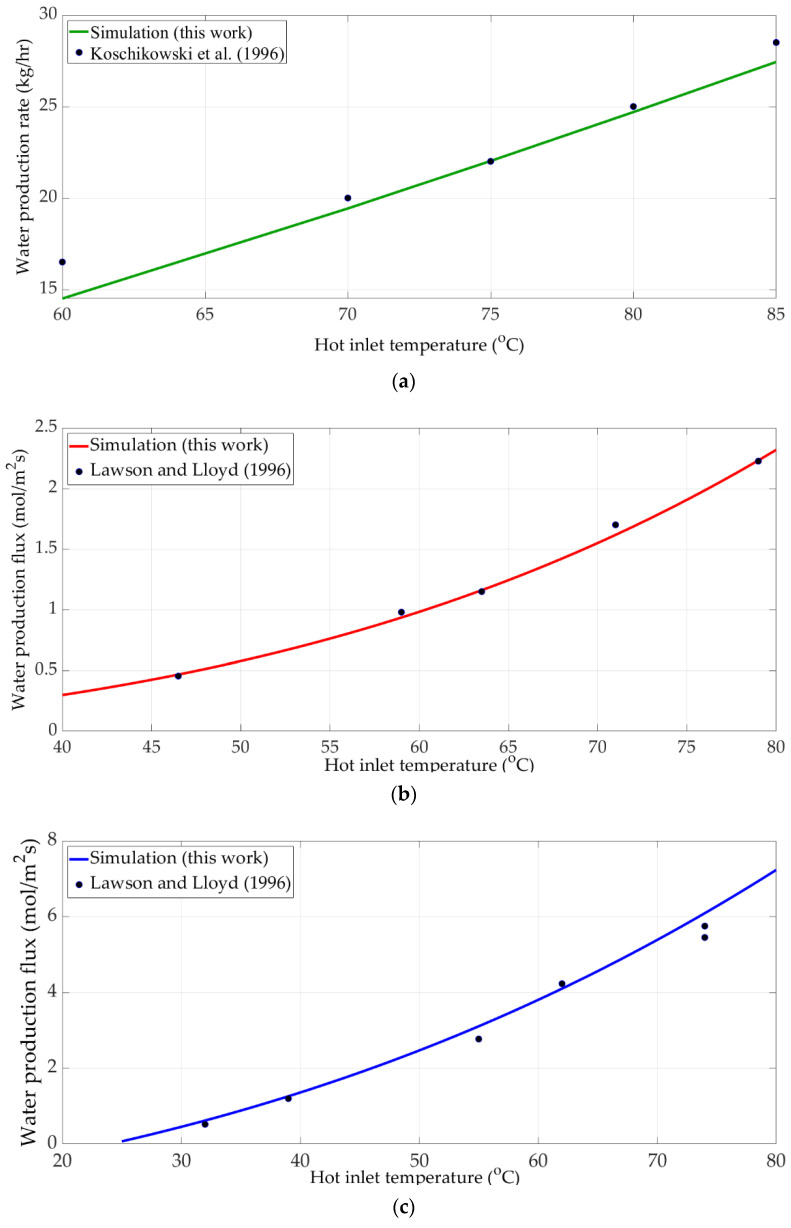
Model validation using experimental data from literature for (**a**) AGMD, (**b**) DCMD, (**c**) VMD modules.

The experimental data of AGMD, DCMD, and VMD modules taken from Koschikowski et al. [30] and Lawson and Lloyd [31,32] were used to verify the membrane distillation models. The properties and materials of the membranes are shown in Table 2. For the DCMD module, membrane with smaller pore size might be needed to prevent operation problems. The smaller pore size gives lower water flux and hence requires higher membrane area for the water production target. The inlet flowrates of the AGMD, DCMD, and VMD modules are 400, 226.8, and 226.8 kg/h, respectively. The cold side temperature of the DCMD module was operated at 20 °C. The vacuum pressure of the VMD module was maintained at 3000 Pa. The mass fluxes of each membrane distillation module were obtained by varying feed temperature, which was operated from 30 to 85 °C. Simulation results and experimental data are compared in Figure 3.

### 2.4. Overall System Simulation

For each of the SDMD system, the unit models illustrated above were developed and simultaneously solved using the solvers provided on Aspen Custom Modeler^®^ (ACM). For each SDMD system, the specifications and simulation results of a representative case are summarized in Table 3.

## 3. Optimization

**Figure 4:**
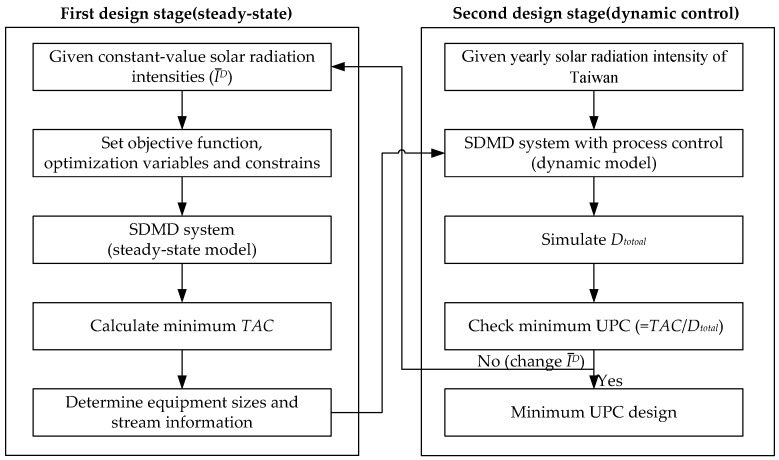
Flow chart of the two-stage optimization.

A two-stage optimization approach for the SDMD system is proposed. The first stage determines optimal equipment sizes and flow rates for each of the specified constant-value solar radiation intensities ( $\bar{I}$^D^) in the studied range. The objective is to minimize the total annual cost (TAC). The second stage is to determine the optimal design for the dynamic operation using the yearly solar radiation intensity of Taiwan. For each of the optimal solution determined from the first stage, the second optimization stage starts by the simulation of that design (equipment sizes) under dynamic operation with a control system proposed by this study. The UPCs (unit production costs) of the distillate water for all the designs from the first stage are then compared to determine the design with the minimum UPC. The two-stage procedure for optimal design of the SDMD system is depicted by the flow chart shown in Figure 4.

Since the system is operated with constant-value solar radiation intensity in the first stage, the optimization analysis requires only steady state simulation. The optimization solver based on the FEASOPT (feasible path successive quadratic programming optimization) method provided in ACM was employed. For the second stage, dynamic simulation must be implemented. The details of the two-stage method are explained in this section.

### 3.1. First Design Stage

#### 3.1.1. Design Variables

For each SDMD system operated under specified constant-value solar radiation intensity, the first design stage is to determine the equipment sizes and the stream operation conditions. The design degree of freedom (DOF) analysis method [33] was used to determine the number of design variables. The design DOFs of the solar-driven AGMD, VMD, and DCMD systems are 10, 11, and 11, respectively. However, considering the manufacture limitations, some dimensions or dimension ratios are specified. The aspect ratio (L_SC_/W_SC_) of the solar collector [34] and the water flow channel thickness in the solar collector (δ_SC_) [35] are set to be 14 and 1 cm, respectively. For all SDMD systems, the aspect ratio of membrane modules (L_MD_/W_MD_) and the flow channel thickness (δ_MD_) of the membrane module [36,37] are set to be 24.44 and 0.63 cm, respectively. The size of storage tank affects the operation temperature. In this study, the tank volume specified for AGMD, DCMD, and VMD systems are 140 m^3^, 153 m^3^, and 225 m^3^, respectively. The ambient temperature (T_a_) and feed temperature of the seawater (T_sea_) are set at 25 °C. The final design DOFs of the solar-driven AGMD, DCMD, and VMD systems are reduced to 5, 6, and 6, respectively. The common design variables of these SDMD systems are constant-value solar radiation intensities ( $\bar{I}$^D^), solar collector area (A_SC_), membrane area (A_MD_), seawater flowrate (F_sea_) and solar collector circulation flowrate (F_sc_). The additional design variables of the DCMD and VMD modules are the MD circulation stream flowrate (F_MD_) and the vacuum pressure (P_v_), respectively.

#### 3.1.2. Cost Functions

The cost functions of the SDMD systems are referred to Banat and Jwaied [16]. Tax rate (i) and the tax amortization period of the asset in years (n) are set to 5% and 15 years, respectively. Based on Banat and Jwaied [16], the cost of solar collector is $100/m^2^. Taking into account the government incentive of $50/m^2^ for solar energy utilization in Taiwan, the cost of solar collector is adjusted to be $50/m^2^. The membrane costs of AGMD, DCMD, and VMD modules are set to $90/m^2^ [16,37]. The capital and operation costs of each unit of the SDMD systems for desalination are shown in Table 4.

#### 3.1.3. Objective Function

The objective of the first design stage is to minimize the TAC of the system. The design DOFs of the SDMD system mentioned in Section 3.1.1 are the decision variables for optimization. The constraints of the optimization problem are:

(1) The distillate water production rate during day-time operation is 2000 kg/h.
(2) The maximum temperature of the hot water from the solar collector is 95 °C.
(3) The minimum approach temperature of the heat exchanger (ΔT_min_) is 10 °C.
(4) The vacuum side pressure of the VMD system is larger than 3 kPa.

For a given constant-value solar radiation intensities ( $\bar{I}$^D^), the optimization problems of the SDMD systems are formulated as:

(1) $$\begin{array}{c} \mathrm{AGMD:}\\ \underset{X\in \Omega }{\mathrm{Minimize}\left(\mathrm{TAC}\right)}\;\Omega =\left\{F_{sea},F_{sc},A_{SC},A_{MD}\right\}.\\ \mathrm{Subject to}\\ T_{\mathrm{sc},\mathrm{out}}\leq 95{\;}^{{}^{\circ}}C,D=2000\frac{kg}{hr},\Delta T_{min}=10{\;}^{{}^{\circ}}C \end{array}$$

(2) $$\begin{array}{c} \mathrm{DCMD:}\\ \underset{X\in \Omega }{\mathrm{Minimize}\left(\mathrm{TAC}\right)}\;\Omega =\{F_{sea},F_{sc},A_{SC},A_{MD},F_{MD}\}\\ \mathrm{Subject}\;\mathrm{to}\\ T_{\mathrm{sc},\mathrm{out}}\leq 95{\;}^{{}^{\circ}}C,D=2000\frac{kg}{hr},\Delta T_{min}=10{\;}^{{}^{\circ}}C \end{array}$$

(3) $$\begin{array}{c} \mathrm{VMD:}\\ \underset{X\in \Omega }{\mathrm{Minimize}\left(\mathrm{TAC}\right)}\;\Omega =\{F_{sea},F_{sc},A_{SC},A_{MD},F_{MD},P_{v}\}\\ \mathrm{Subject}\;\mathrm{to}\\ T_{\mathrm{sc},\mathrm{out}}\leq 95{\;}^{{}^{\circ}}C,D=2000\frac{\mathrm{kg}}{h},\Delta T_{min}=10{\;}^{{}^{\circ}}C,P_{v}\geq 3\;\mathrm{kPa} \end{array}$$

### 3.2. Second Design Stage

#### 3.2.1. Control System Design

**Figure 5:**
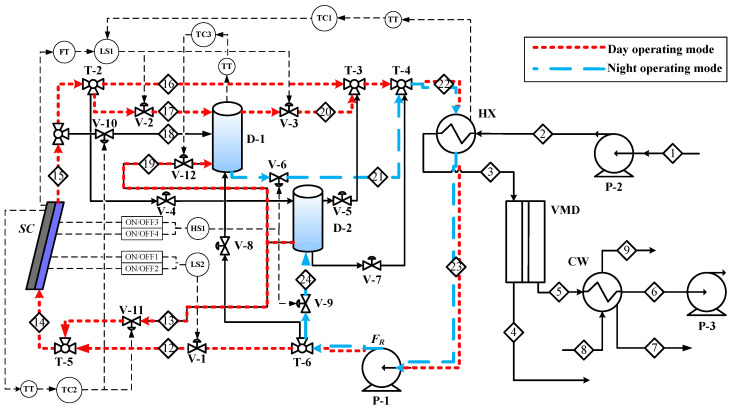
The control structure of the solar-driven VMD system for desalination.

Based on the control structure designed for maintaining the desalinated water production rate of the solar-driven AGMD system with unpredictive solar energy intensity developed by the authors [20], modified control structures were developed of the solar-driven membrane distillation systems for desalination. The control structure of the solar subsystem is the same for the solar-driven AGMD, DCMD, and VMD systems, hence only the control structure of the solar-driven VMD system is explained and illustrated in Figure 5.

The thermal storage tank is used as a heat sink to collect solar energy by using a solar collector and to adjust the supply of the unpredictive solar energy. Water is used as the working fluid to transfer the solar energy from the solar collector to the thermal storage tank. The hot water from the thermal storage tank then provides the heat to the membrane distillation subsystem for desalination. In the day-time operation mode (red line in Figure 5), the hot water is supplied from the thermal storage tank (D-1) and the returning water from the heat exchanger (HX) (stream 23) is recycled back to the solar collector (SC) for absorbing the solar energy and stored in D-1. During the night-time operation (blue line in Figure 5), the hot water is also supplied from D-1 but the returning water is directly returned to D-2 to prevent the temperature decrease in D-1. The main control loops of the solar-driven VMD system include:

(1) The temperature (T_22_) of the hot water entering the heat exchanger (HX-1) is controlled by manipulating the inlet flowrate (F_17_) of D-1. The purpose of this control loop is to maintain constant water production rate.
(2) The outlet stream temperature (T_15_) of the solar collector is controlled at 95 °C by manipulating the make-up water flowrate (F_13_) from D-2. The control loop was used to avoid the working fluid boiling problem.
(3) The make-up water flow (F_19_) from D-2 to the thermal storage tank is used to maintain the temperature of the thermal storage tank (T_20_) below T_22_. As the temperature control loop of T_22_ cannot work when T_20_ approaches T_22_.

The Auto-tuning variation method (ATV) [39,40] was employed for the tuning of controller parameters. The T-L tuning rule is used to calculate the controller gain (K_C_) and integral time (τ_I_) for the PI temperature controllers.

#### 3.2.2. Objective Function

The objective function in the second design stage is the unit production cost (UPC = TAC/D_total_), which is obtained by dividing the TAC to the annual production rate of water. The distillate water can be blended with raw water to provide potable water. The dilution ratio is the ratio of the distillate water to the raw water. Here, the dilution ratio of the water production is set at 1:1 [16]. For each SDMD system using the equipment sizes determined from the first stage for different constant-value solar radiation intensities ( $\bar{I}$^D^) and operated with the control structure shown in Figure 5 and annual solar radiation intensity of Taiwan, the annual water production rate can be evaluated by using the dynamic simulation model presented in Section 2.2.

## 4. Results and Discussion

### 4.1. Optimal Solutions from the First Design Stage

**Figure 6:**
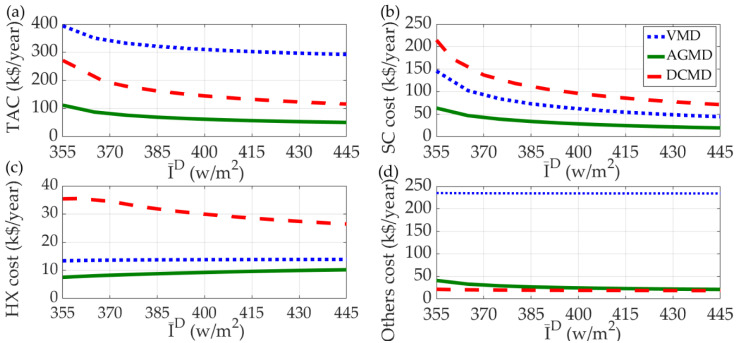
Optimal solutions of solar-driven AGMD, DCMD, and VMD systems for desalination from the first design stage for different constant-value solar radiation intensity, (**a**) TAC, (**b**) solar collector cost, (**c**) heat exchanger cost, (**d**) cost of all other units.

For constant-value solar radiation intensities ($\bar{I}$^D^) ranged from 355 to 445 W/m^2^, the optimal equipment sizes and stream flow rates are determined from the first optimization stage. The TAC and the costs of SC, HX, and all other units of the optimal solutions are shown in Figure 6.

In Figure 6a, it is shown that the TACs of the SDMD systems employing AGMD, DCMD, and VMD are all decrease with the increase of $\bar{I}$^D^ and the highest and the lowest TAC are the systems employing VMD and AGMD, respectively. The effect of $\bar{I}$^D^ on the cost of SC follows the same trend with TAC as shown in Figure 6b. However, the DCMD system requires the highest SC cost and the lowest cost is still the AGMD system. Regarding the cost of heat exchanger, as shown in Figure 6c, the highest is the DCMD system and decreases with the increase of $\bar{I}$^D^. However, the heat exchanger costs of both VMD and AGMD systems show slight increase with the increase of $\bar{I}$^D^ and the lowest is the AGMD system. As shown in Figure 6d, $\bar{I}$^D^ shows only minor effect on the cost of all other equipment units for the three systems. The cost of all other units of the VMD system is significantly higher than the other two systems.

### 4.2. Optimal Solutions from the Second Design Stage

**Figure 7:**
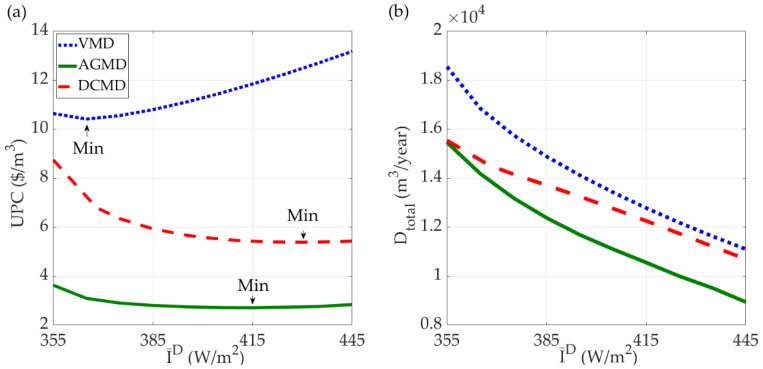
The (**a**) UPC and (**b**) annual water production rate of solar-driven MD systems under dynamic control with yearly solar radiation intensity of Taiwan. The optimal equipment sizes determined from the first design stage for each $\bar{I}$^D^ are used.

The simulation results of UPC and annual water production rate (D_total_) under dynamic control for the three SDMD systems are shown in Figure 7. Note that the optimal equipment sizes determined from the first design stage for each $\bar{I}$^D^ are used.

The results shown in Figure 7a indicate that the UPCs of the VMD system are the highest and the AGMD system gives the lowest UPCs. For each system, the design with the minimum UPC can be identified from the optimization analysis. The results in Figure 7b indicate that the water production rates of the three systems all decrease with the increase of $\bar{I}$^D^. The explanation is that the optimal size of solar collector is smaller when $\bar{I}$^D^ is higher, which has been shown in Figure 6b. Consequently, less thermal energy can be stored during the daytime for night-time operation, which hence leads to a lower total production rate of water. The minimum UPC designs marked in Figure 7a are resulted from the trade-off of TAC and D_total_, which are shown in Figure 6a and Figure 7b, respectively.

The minimum UPC designs shown in Figure 7a for each of the SDMD system are summarized in Table 5. The solar collector area of the AGMD system is only 32% and 20% of the DCMD system and VMD system, respectively. On the contrary, the membrane area of the AGMD system is 2.75 and 8.41 times of the DCMD system and the VMD system, respectively. For the AGMD, DCMD, and VMD systems, the optimal unit water production costs are $2.71/m^3^, $5.38/m^3^, and $10.41/m^3^, respectively. The results indicate that the AGMD system is the most economic and its UPC is about 50% and 26% of the DCMD system and VMD system, respectively.

### 4.3. Comparison of Costs of SDMD Systems

**Figure 8:**
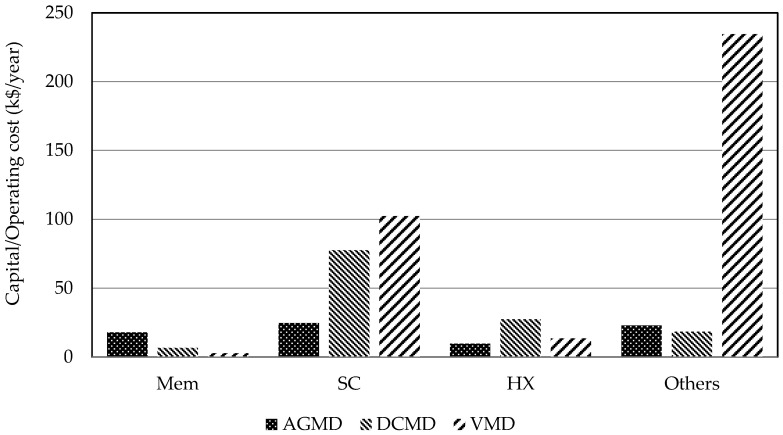
Equipment costs of the optimal SDMD systems for desalination.

The main equipment of the SDMD systems for desalination are membrane distillation modules, heat exchanges, and solar collectors. The equipment costs of the optimal SDMD systems for desalination are compared in Figure 8. The comparison of the membrane distillation module costs indicates AGMD > DCMD > VMD and that of the solar collector costs indicates VMD > DCMD > AGMD. Regarding the cost of the heat exchanger, the DCMD system is the highest and the VMD system is the second highest. These results of the costs of membrane distillation module and solar collector can be explained by the features of the three types of membrane distillation modules. For AGMD, the air gap gives extra mass transfer resistance and results in larger membrane area. For DCMD, the fluids in contact with the membrane are both liquid phase. That causes greater energy loss via heat conduction and results in larger solar collector area and heat exchanger area. For VMD, the vacuum operation significantly enhances the mass flux across the membrane and results in the smallest membrane area but the largest solar collector area. The cause of the high heat exchanger cost of DCMD is the extra heat exchanger for the cold liquid circulation of the membrane distillation module and the high circulation rate of that stream. The large solar collector area of VMD requires high hot water circulation flow rate as well as high heat exchange area.

### 4.4. Effect of Membrane Cost

**Figure 9:**
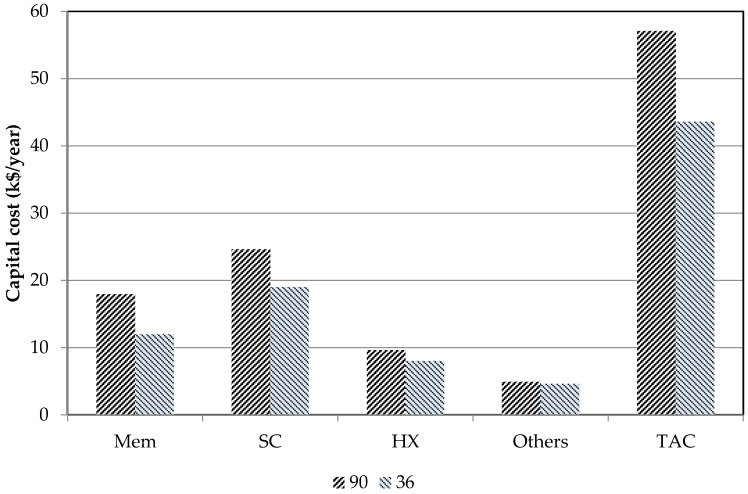
Equipment costs of the optimal solar-driven AGMD system for desalination with different membrane costs ($90/m^2^ and $36/m^2^).

**Figure 10:**
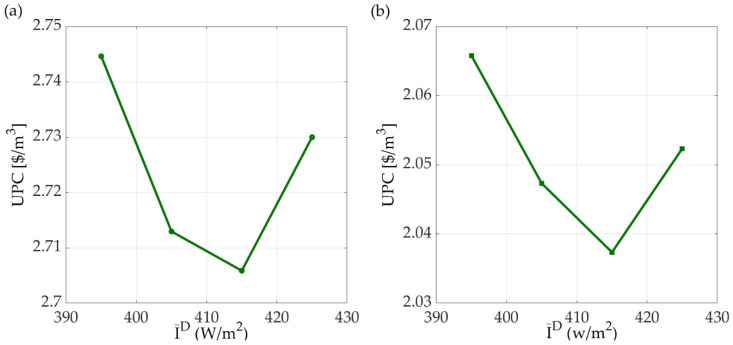
The unit water production cost of the solar-driven AGMD system for desalination with a membrane unit cost of (**a**) $90/m^2^ (**b**) $36/m^2^.

In this work, the membrane unit cost is set to $90/m^2^ [32]. Sensitivity of the optimal design to the membrane unit cost was analyzed for the most economical AGMD system. When the membrane unit cost is reduced to $36/m^2^ [16], the optimal design results of the solar-driven AGMD system are different. The equipment costs of the optimal solar-driven AGMD system using different membrane unit costs are compared in Figure 9. When the membrane unit cost is lower, larger membrane distillation module and smaller sizes of other equipment units are used. The costs of all types of equipment units as well as the total annual cost are reduced. The UPC results of the solar-driven AGMD system are compared in Figure 10. When the membrane unit cost is reduced from $90/m^2^ to $36/m^2^, the UPC of the solar-driven AGMD system for desalination reduced from $2.71/m^2^ to $2.04 m^2^. This result indicates that the membrane unit cost does not significantly affect the optimal design and the UPC of the solar-driven membrane distillation systems for desalination.

## 5. Conclusions

In this work, the solar-driven membrane distillation (SDMD) systems utilizing AGMD, DCMD, and VMD for desalination are investigated. The Aspen Custom Molder^®^ (ACM) simulator is used to build and solve for the steady state and dynamic models of the SDMD systems. The simulation results of the membrane distillation models fit well with the literature experimental data. For better operation of the system to cope with the unpredictive and intermittent characteristics of solar radiation, a control structure was designed for the solar subsystem.

Optimal designs of the SDMD systems were determined by the two-stage design approach proposed in this study. The first stage design determines the equipment sizes for constant-value solar radiation intensity using the steady state model. The second stage analyzes the operation with the control structure for the yearly solar radiation intensity of Taiwan using the dynamic model. The comparison of the optimal designs of the solar-driven AGMD, DCMD, and VMD systems indicates that the AGMD system requires the largest membrane distillation module, the DCMD system calls for the largest heat exchanger, and the VMD system needs the largest solar collector. The unit production costs (UPCs) of the optimal solar-driven AGMD, DCMD, and VMD systems for desalination are $2.71/m^3^, $5.38/m^3^, and $10.41/m^3^, respectively. Both TAC and UPC of the solar-driven AGMD system are the lowest. The effects of the membrane unit cost on the optimal design and the water production cost are not significant. With a decrease of membrane unit cost from $90/m^2^ to $36/m^2^, the UPC of the optimal solar-driven AGMD system reduces from $2.71/m^2^ to $2.04/m^2^.

## Figures and Tables

**Table 1:** Model equations for each unit of solar-driven MD systems for desalination.

**1. Solar subsystem**
(1) Solar collector
$\frac{dT_{c}}{dt}=\frac{AU}{M_{c}C_{p,c}}\left(\frac{BI\left(t\right)}{U}+T_{a}\left(t\right)-T_{c}\right)-\frac{Ah}{M_{c}C_{p,c}}\left(T_{c}-T_{f}\right)$
$\frac{\partial T_{f}}{\partial t}=-L\frac{m_{f,c}}{M_{f}}\frac{\partial T_{f}}{\partial z}+\frac{Ah}{M_{f}C_{p,w}}\left(T_{c}-T_{f}\right)$
(2) Heat exchanger
$\frac{\partial T_{hl}}{\partial t}=L\frac{m_{hl}}{M_{hl}}\left(\frac{\partial T_{hl}}{\partial x}\right)-\frac{A_{HX}U}{M_{hl}C_{phl}}\left(T_{hl}-T_{cl}\right)$
$\frac{\partial T_{cl}}{\partial t}=L\frac{m_{cl}}{M_{cl}}\left(\frac{\partial T_{cl}}{\partial x}\right)+\frac{A_{HX}U}{M_{cl}C_{pcl}}\left(T_{hl}-T_{cl}\right)$
(3) Storage tank
$\frac{dM}{dt}=m_{f,in}-m_{f,out}$
$\frac{dT_{w}}{dt}=\frac{m_{f}\left(T_{1}-T_{w}\right)+m_{f,in}\left(T_{2}-T_{w}\right)}{M}$
**2. AGMD subsystem**
$m_{f,hl}\left(k\right)=m_{f,hl}\left(k-1\right)-N_{gm}\left(k\right)A\left(k\right)$
$N_{gm}\left(k\right)=\frac{k_{gm}}{\frac{R\left(T_{gm1}\left(k\right)+T_{gm2}\left(k\right)\right)}{2}}\left(P_{gm1}\left(k\right)-P_{ag}\left(k\right)\right)$
$m_{f,conl}\left(k\right)=m_{f,conl}\left(k-1\right)+N_{ag}\left(k\right)A\left(k\right)$
$N_{ag}\left(k\right)=\frac{k_{ag}P_{ag}^{T}}{\frac{RP_{In\;air}\left(k\right)\left(T_{gm2}\left(k\right)+T_{conl}\left(k\right)\right)}{2}}\left(P_{ag}\left(k\right)-P_{conl}\left(k\right)\right)$
$N_{hl}\left(k\right)A\left(k\right)=N_{gm}\left(k\right)A\left(k\right)=N_{ag}\left(k\right)A\left(k\right)$ $Q_{hl}\left(k\right)=\left(h_{hl}\left(k\right)+N_{hl}\left(k\right)C_{p\;hl}\right)\left(T_{hl}\left(k\right)-T_{gm1}\left(k\right)\right)$
$Q_{hl}\left(k\right)A\left(k\right)=\left(Q_{gm}\left(k\right)+N_{gm}\left(k\right)h_{vap,gm1}\left(k\right)\right)A\left(k\right)$
$Q_{gm}\left(k\right)=\left(h_{gm}\left(k\right)+N_{gm}\left(k\right)C_{p\;gm}\right)\left(T_{gm1}\left(k\right)-T_{gm2}\left(k\right)\right)$
$Q_{gm}\left(k\right)A\left(k\right)=Q_{ag}\left(k\right)A\left(k\right)$
$Q_{gm}\left(k\right)=\left(h_{ag}\left(k\right)+N_{gm}\left(k\right)C_{p\;ag}\right)\left(T_{gm2}\left(k\right)-T_{conl}\left(k\right)\right)$
$\left(Q_{ag}\left(k\right)+N_{gm}\left(k\right)h_{vap,conl}\left(k\right)\right)A\left(k\right)=Q_{conl}\left(k\right)A\left(k\right)$
$Q_{conl}\left(k\right)=\left(h_{conl}+N_{ag}\left(k\right)C_{p\;conl}\right)\left(T_{conl}\left(k\right)-T_{m1}\left(k\right)\right)$
$Q_{m}\left(k\right)=h_{m}\left(T_{m1}\left(k\right)-T_{m2}\left(k\right)\right)$
$Q_{conl}\left(k\right)A\left(k\right)=Q_{m}\left(k\right)A\left(k\right)$
$Q_{cl}\left(k\right)=h_{cl}\left(T_{m2}\left(k\right)-T_{d}\left(k\right)\right)$
$Q_{m}\left(k\right)A\left(k\right)=Q_{cl}\left(k\right)A\left(k\right)$
$k_{gm}=\frac{\varepsilon }{\tau \delta_{m}}\left(\frac{1}{\frac{1}{D_{k}}+\frac{y_{air,lm}}{D_{m}}}\right)$
$D_{k}=\frac{2}{3}\frac{\varepsilon r}{\tau \delta_{m}}M_{w}\sqrt{\frac{8RT_{avg.}}{\pi M_{w}}}$
$K_{ag}=\frac{D_{m}}{\delta_{ag}}$
$D_{m}=\frac{1.43\times 10^{-7}\times {T_{avg.}}^{1.75}}{\frac{P_{system}}{101325}\times {M_{w,air}}^{0.5}\times {\left(\delta^{\frac{1}{3}}{}_{N2}+\delta^{\frac{1}{3}}{}_{H_{2}O}\right)}^{2}}$
hi(k)=0.065Re(k)0.875Pr(k)0.25kcDh
Re(k)=ρuDhμ(k)
$\mathrm{Pr}\left(k\right)=\frac{\mu \left(k\right)C_{p}}{k_{c}M_{w}}$
$\mu \left(k\right)=0.001{\left(0.9^{-0.2661\frac{25-T_{i}\left(k\right)}{233}}\right)}^{-\frac{1}{0.2661}}$
$P_{i}\left(k\right)=\exp\left(72.55-\frac{7206.7}{T_{i}\left(k\right)}-7.1385\times \ln(T_{i}\left(k\right)\right)+\left({4.046\times 10}^{-6}\right)\times T_{i}{\left(k\right)}^{2})$
**3. DCMD subsystem**
$m_{f,hl}\left(k\right)=m_{f,hl}\left(k-1\right)-N_{gm}\left(k\right)A\left(k\right)$
$N_{gm}\left(k\right)=\frac{\frac{k_{gm}}{R\left(T_{gm1}\left(k\right)+T_{gm2}\left(k\right)\right)}}{2}\left(P_{gm1}\left(k\right)-P_{gm2}\left(k\right)\right)$
$N_{hl}\left(k\right)A\left(k\right)=N_{gm}\left(k\right)A\left(k\right)=N_{cl}\left(k\right)A\left(k\right)$
$m_{f,cl}\left(k\right)=m_{f,cl}\left(k-1\right)+N_{gm}\left(k\right)A\left(k\right)$
$Q_{hl}\left(k\right)=\left(hl_{hl}\;\left(k\right)+N_{hl}\left(k\right)C_{P\;hl}\right)\left(T_{hl}\left(k\right)-T_{gm1}\left(k\right)\right)$
$Q_{hl}\left(k\right)A\left(k\right)=\left(Q_{gm}\left(k\right)+N_{gm}\left(k\right)h_{vap,gml}\left(k\right)\right)A\left(k\right)$
$Q_{gm}\left(k\right)=(h_{gm}\left(k\right)+N_{gm}\left(k\right)C_{p\;gm})\left(T_{gm1}\left(k\right)-T_{gm2}\left(k\right)\right)$
$\left(Q_{m}\left(k\right)+N_{gm}\left(k\right)h_{vap,gm2}\left(k\right)\right)A\left(k\right)=Q_{cl}\left(k\right)A\left(k\right)$
$Q_{cl}\left(k\right)=\left(h_{cl}\left(k\right)+N_{cl}\left(k\right)C_{p,cl}\right)\left(T_{gm2}\left(k\right)-T_{cl}\left(k\right)\right)$
hi(k)=1.86khl(k)(Re(k)Pr(k)Dh 2L)0.33 (Re≤2100)
$h_{i}\left(k\right)=0.023k_{hl}\left(k\right)\frac{Re{\left(k\right)}^{0.8}\mathrm{Pr}{\left(k\right)}^{0.33}}{D_{h}}\;\left(Re>2100\right)$
**4. VMD subsystem**
$m_{f,hl}\left(k\right)=m_{f,hl}\left(k-1\right)-N_{gm}\left(k\right)A\left(k\right)$
$N_{gm}\left(k\right)=\frac{\frac{k_{gm}}{R\left(T_{gm1}\left(k\right)+T_{gm2}\left(k\right)\right)}}{2}\left(P_{gm1}\left(k\right)-P_{v}\left(k\right)\right)$
$N_{hl}\left(k\right)A\left(k\right)=N_{gm}\left(k\right)A\left(k\right)=N_{v}\left(k\right)A\left(k\right)$
$m_{f,v}\left(k\right)=m_{f,v}\left(k-1\right)+N_{gm}\left(k\right)A\left(k\right)$
$Q_{hl}\left(k\right)=\left(h_{hl}\left(k\right)+N_{hl}\left(k\right)C_{P\;hl}\right)\left(T_{hl}\left(k\right)-T_{gm1}\left(k\right)\right)$
$Q_{hl}\left(k\right)A\left(k\right)=\left(Q_{gm}\left(k\right)+N_{gm}\left(k\right)h_{vap,gm1}\left(k\right)\right)A\left(k\right)$
$Q_{gm}\left(k\right)=\left(h_{gm}\left(k\right)+N_{gm}\left(k\right)C_{p\;gm}\right)\left(T_{gm1}\left(k\right)-T_{gm2}\left(k\right)\right)$
$Q_{gm}\left(k\right)A\left(k\right)=Q_{v}\left(k\right)A\left(k\right)$
$k_{gm}=\frac{1}{\delta_{m}}\left[K_{0}\sqrt{\frac{8RT_{avg.}}{\pi M_{w}}}+B_{0}\frac{P_{avg.}\left(k\right)}{\mu (k)}\right]$

**Table 2:** Membrane information [30,31,32].

Property	AGMD	DCMD	VMD
Material	PTFE	PP(3ME)	PP(3MA)
Pore size(γ) (μm)	0.200	0.730	0.290
Porosity(ε)	0.770	0.850	0.660
Thickness(δ) (mm)	0.140	0.079	0.091
Area(A_MD_) (m^2^)	7.000	9.7 × 10^−4^	9.7 × 10^−4^
Aspect ratio (L/W)	0.070	24.440	24.440

**Table 3:** The equipment sizes and operation conditions of a representative case for each SDMD system.

Specifications and Results	AGMD	DCMD	VMD
Solar collector area (A_SC_) (m^2^)	1615.00	18,350.00	10,297.00
Membrane area (A_MD_) (m^2^)	2040.00	500.00	58.00
Heat transfer area (A_HX_) (m^2^)	100.00	4000.00	380.00
Constant-value solar radiation intensity ($\bar{I}$^D^) (W/m^2^)	458.75	458.75	458.75
Solar collector circulation flowrate (F_sc_) (kg/h)	24,940	45,350.00	24,680.00
Solar collector outlet temperature (T_sc,out_) (°C)	84.38	93.55	94.82
Seawater flowrate (F_sea_) (kg/h)	30,434.00	40,100.00	32,545.00
MD circulation stream flowrate (F_MD_) (kg/h)	-	50,000.00	-
MD inlet stream temperature (T_MD,in_) (°C)	75.57	92.99	70.36
Vacuum pressure (P_v_) (Pa)	-	-	5000.00
Water production rate (D) (kg/h)	2000.00	2000.00	2000.00

**Table 4:** Capital and operation costs of each unit of the SDMD system for desalination [38].

Amortization factor, a
$a=\frac{i{\left(1+i\right)}^{n}}{{\left(1+i\right)}^{n}-1}$
Capital cost of centrifugal pumps ($)
S = Q(H)^0.5^
C_B_ = exp{9.7171 − 0.6019[ln(S)] + 0.0519[ln(S)]^2^}
C_P_ = F_T_F_M_C_B_
Capital cost of vacuum pumps ($)
S = 50-350 (ft^3^/min)
C_p_ = 8250S^0.35^
Capital cost of shell and tube heat exchangers ($)
C_B_ = exp{11.0545 − 0.9228[ln(A)] + 0.09861[ln(A)]^2^}
F_M_ = a + (A/100)^b^
F_P_ = 0.09803 + 0.018(P/100) + 0.0017(P/100)^2^
F_L_ = 1
C_P_ = F_P_ F_M_ F_L_C_B_
Capital cost of porous membranes ($)
C_p_ = 90 A_m_
Replacement cost of porous membranes ($/yr)
C_p, replacement_ = C_p, i_ * 20% i = AGMD, DCMD or VMD
Capital cost of solar collector ($)
C_p_ = 50 A_SC_
Utility unit cost
Electricity cost: $0.06/kWh

**Table 5:** Optimal design of the SDMD systems for desalination.

Variables	AGMD	DCMD	VMD
Constant-value solar radiation intensities ($\bar{I}$^D^) (W/m^2^)	415.00	430.00	360.00
Solar collector area (A_SC_) (m^2^)	5111.00	16,104.00	26,066.00
Membrane area (A_MD_) (m^2^)	673.00	245.00	80.00
Heat transfer area (A_HX_) (m^2^)	116.00	546.00	274.00
Solar collector circulation flowrate (F_sc_) (kg/h)	22,854.00	34,984.00	21,446.00
Solar collector outlet temperature (T_sc,out_) (°C)	95.00	95.00	95.00
Seawater flowrate (F_sea_) (kg/h)	28,657.00	30,142.00	35,302.00
MD circulation stream flowrate (F_MD_) (kg/h)	-	48,183.00	-
Vacuum pressure (P_v_) (Pa)	-	-	3554.00
Annual water production rate (D_total_) (m^3^/year)	10,550.00	11,470.00	16,854.00
Total annual cost (TAC) ($/year)	57,092.00	123,411.00	350,565.00
Unit production cost (UPC) ($/m^3^)	2.71	5.38	10.41

## Data Availability

Data sharing is not applicable to this article.

## Nomenclature

A	Area (m^2^)
a	Amortization factor
B	Absorption coefficient (-)
b_f_	Channel height of the membrane distillation module (m)
b_sc_	Channel height of the solar collector (m)
C_B_	Bare module cost ($)
C_p_	Purchase cost ($)
C_pm_	Heat capacity (J/kg K)
C_pc_	Heat capacity of the solar collector plate (J/kg K)
C_pf_	Heat capacity of the working fluid (J/kg K)
D	Water production rate (kg/h)
D_h_	Hydraulic diameter (m)
D_total_	Annual water production rate (m^3^/year)
F_L_	Piping length factor
F_M_	Material of construction factor
F_MD_	MD circulation stream flowrate (kg/h)
F_P_	Pressure factor
F_sea_	Seawater flowrate (kg/h)
F_SC_	Solar collector circulation flowrate (kg/h)
F_T_	Pump type factor
FT	Flow transmitter
H	Head (m)
HS	High signal selector
HX	Heat exchanger
h	Heat convention coefficient (W/m^2^ K)
h_vap_	Heat of vaporization (J/kmol)
$\bar{I}$ ^D^	Constant-value solar radiation intensities (W/m^2^)
i	Tax rate (%)
K_C_	Controller gain (%/%)
k	Heat conduction coefficient (J/s m^2^ K)
L	Length (m)
LS	Low signal selector
L/W	Aspect ratio (-)
M	Weight (kg)
mw	Molecular weight (kg/kmol)
m	Mass flowrate (kg/h)
N	Molar flux (kmol/m^2^ s)
n	Tax amortization period of the asset in years
P	Pressure (Pa)
Q	Heat flux (kJ/m^2^s)
S	Equipment size coefficient (-)
T	Temperature (°C)
TAC	Total annual cost ($/yr)
TC	Temperature control
TT	Temperature transmitter
U	Overall heat transfer coefficient (W/m^2^ K)
UPC	Unit production cost($/m^3^)
V	Velocity (m/s)
W	Width (m)
Symbol	
ΔT_min_	Minimum approach temperature of the heat exchanger (°C)
γ	Pore size (μm)
δ	Thickness (μm)
ε	Porosity of the membrane (-)
τ_I_	Integral time
Subscript	
a	Ambient
ag	Air gap layer
cl	Cold fluid
conl	Condensate fluid
f	Working fluid
gm	Internal layer of the membrane
gm1	Interfacial between the hot fluid and the membrane
gm2	Interfacial between the membrane and the other fluid
HX	Heat exchanger
hl	Hot fluid
in	Inlet
MD	Membrane
m	Metal layer
met1	Interfacial between the metal and the air gap
met2	Interfacial between the metal and the cold fluid
No.	Stream no.
o	Outlet
SC	Solar collector
sea	Seawater flowrate
v	Vacuum
w	Storage tank
